# Dynamic Behavior of Advanced Materials and Structures (Second Edition)

**DOI:** 10.3390/ma19143000

**Published:** 2026-07-12

**Authors:** Weidong Song, Lijun Xiao

**Affiliations:** State Key Laboratory of Explosion Science and Safety Protection, Beijing Institute of Technology, Beijing 100081, China; xljbit@bit.edu.cn

Driven by the growing demands for lightweight, high-strength, and energy-absorbing systems in aerospace, defense, automotive, and civil infrastructure, studies on the dynamic behavior of advanced materials and structures have become increasingly crucial [1,2,3]. Under high strain rate loading conditions such as high-velocity impacts, blast loads, and ballistic penetration, materials and structures exhibit complex rate-dependent deformation, damage evolution, and failure mechanisms that differ significantly from those under quasi-static or low-rate loading [4,5,6,7]. This Special Issue (SI), “Dynamic Behavior of Advanced Materials and Structures (Second Edition)”, presents recent theoretical, experimental, and simulation research findings regarding the dynamic behavior of advanced material and structures. This Editorial summarizes the nine publications included in this SI.

As typical reactive materials, Polytetrafluoroethylene/aluminum (PTFE/Al) composite can be triggered to react and release substantial chemical energy under intense shock loading [8,9,10]. Tian et al. [11] examined the reaction mechanism of PTFE/Al composite under shock compression, revealing that when the shock compression intensity drops from GPa to MPa levels, the reaction ignition and energy release of PTFE/Al materials transition from immediately following the shock front to lagging behind it by hundreds of microseconds.

Porous materials present excellent energy absorption capacity and are widely applied in anti-impact scenarios [12,13,14]. Xie et al. [15] investigated the effect of the pore shape and size on the failure mechanism of the porous materials. The results suggested that two distinct dominant deformation modes existed for differently shaped pores, and that increasing the pore size could shift the model from strain-hardening to quasi-zero stiffness. Song et al. [16] adopted the bidirectional evolutionary structural optimization (BESO) method to design lattice structures with maximum bulk modulus and elastic isotropy, verifying the superior mechanical properties of the topology-optimized lattices over traditional lattices in terms of modulus, yield strength, and specific energy absorption. Xiao et al. [17] explored the dynamic performance of 3D-printed short fiber-reinforced polymer (SFRP) porous structures under low speed impact. They concluded that competition between damage evolution and strain rate strengthening significantly affected the energy absorption efficiency of the structures, while the structural instability was dominated by the initiation and propagation of shear bands.

In aerospace service environments, the coupling effect between overload and fiber bridging makes it very difficult to predict the crack propagation behavior of fiber metal laminates [18,19]. Meng et al. [20] combined the experimental results with the improved equivalent crack length model, proposing the prediction models for the crack propagation behavior of Fiber/Al-Li laminates under single-peak tensile and compressive overloads, respectively. The predicted results by these models were in excellent agreement with the experimental data.

As the dynamic response of aluminum alloys across a wide strain-rate range remain poorly understood, their application in lightweight armor systems faces great limitations [21]. Olasumboye et al. [22] discussed the effects of heat treatment on the microstructure and dynamic deformation characteristics of AA2519 aluminum alloy within a wide strain rate range from 1000 s^−1^ to 4000 s^−1^. They revealed that different temper conditions resulted in various deformation mechanisms and mechanical properties.

Resilient mounts are widely used in vibration reduction and shock absorption systems, which makes it quite crucial to accurately predict their static and dynamic behaviors [23]. Park et al. [24] calibrated the parameters of the Yeoh hyperelastic model through experiments to describe the quasi-static mechanical behavior of the resilient mount. Combined with modal analysis and frequency response simulations under various preload conditions, the dynamic behavior of the resilient mount was also analyzed. The results indicated that increasing the preload significantly shifted the transmissibility curves and resonance peaks to lower frequencies.

The elastic ring squeeze film damper (ERSFD) is a key component in various types of combination bearings for aero-engines [25,26]. Zhang et al. [27] proposed an equivalent stiffness method for the elastic ring and conducted parametric analyses on the pressure distribution of the inner and outer oil films, providing a theoretical basis for the design, application, and maintenance of combination bearings incorporating elastic ring squeeze film dampers.

Due to differences in aggregate gradation and construction-induced interfaces, hydraulic concrete exhibits distinct dynamic mechanical behaviors from those of ordinary concrete [28]. Wang et al. [29] investigated the effects of aggregate gradation and bonding interfaces on the dynamic failure modes of hydraulic concrete through mesoscale numerical simulations, revealing the strain rate sensitivity of the dynamic tensile and compressive strengths of hydraulic concrete.

It should be noted that in realistic service environments, materials and structures often experience the coupling of multiple physical effects when subjected to extreme loads: for example, high-velocity impact combined with high temperatures, phase transitions, or electromagnetic effects [30,31,32,33]. Understanding and accurately simulating these multiphysics effects remains a great challenge. Moreover, the emergence of artificial intelligence (AI) offers transformative opportunities for the field. Machine learning techniques can accelerate the discovery of constitutive models [34], enable real-time impact response prediction [35,36], optimize structural designs for energy absorption [37], and extract hidden patterns from high-dimensional experimental or simulation data, which may enable new frontiers to be addressed in impact protection and material design.
